# Silver Nanoparticles Used in Medical–Dental Plastics for Therapeutic Purposes: A Comprehensive Review

**DOI:** 10.3390/antibiotics14121267

**Published:** 2025-12-15

**Authors:** José Luis Maruri-Casas, Edith Lara-Carrillo, Víctor Hugo Toral-Rizo, Raúl Alberto Morales-Luckie, Gloria Elena Guzmán-Celaya, Norma Guadalupe Ibañez-Mancera, Francisco Javier Tejeda-Nava, Saraí Carmina Guadarrama-Reyes, Elías Nahúm Salmerón-Valdés, Ana Miriam Santillán-Reyes

**Affiliations:** 1 Doctoral Student in Health Sciences Program, Research Center and Advanced Studies in Dentistry “Dr. Keisaburo Miyata”, Faculty of Dentistry, Autonomous University of the State of Mexico, Toluca 50130, Mexico; jmaruric077@alumno.uaemex.mx; 2 Department of Orthodontics, Research Center and Advanced Studies in Dentistry “Dr. Keisaburo Miyata”, Faculty of Dentistry, Autonomous University of the State of Mexico, Toluca 50130, Mexico; 3 Head of the Department of Oral Medicine and Bucal Pathology, Faculty of Dentistry, Autonomous University of the State of Mexico, Toluca 50130, Mexico; 4 Department of Materials Science, Center for Research in Sustainable Chemistry, Autonomous University of the State of Mexico, Toluca 50200, Mexico; ramoralesl@uaemex.mx; 5 Faculty of Dentistry, Autonomous University of Sinaloa, Culiacán 80010, Mexico; gloriaguzman@uas.edu.mx; 6 Interdisciplinary Center for Health Sciences CICS-UST, National Polytechnic Institute, Mexico City 11340, Mexico; nibanezm@ipn.mx; 7 Faculty of Dentistry, Autonomous University of San Luis Potosí, San Luis Potosí 78290, Mexico; francisco.tejeda@uaslp.mx; 8 Faculty of Dentistry, Autonomous University of the State of Mexico, Toluca 50130, Mexico; scguadarramar@uaemex.mx (S.C.G.-R.); ensalmeronv@uaemex.mx (E.N.S.-V.); amsantillanr@uaemex.mx (A.M.S.-R.)

**Keywords:** silver nanoparticles, plastics, biomaterials, antimicrobial, dentistry, medicine

## Abstract

Background/Objectives: The integration of silver nanoparticles (AgNPs) into plastic materials has emerged as a promising strategy to provide biomedical and dental devices with an active antimicrobial barrier. In this review, we aimed to synthesize the scientific evidence published between 2013 and 2024 regarding the therapeutic efficacy, biocompatibility, and safety of AgNP-functionalized plastics by following the 2020 PRISMA guidelines. Methods: Searches were conducted in PubMed, Scopus, Web of Science, SciELO, and ScienceDirect. A total of 634 records were identified, of which 21 studies met the inclusion criteria after full-text evaluation. Results: Plastics containing AgNPs show a significant reduction in microbial load (*Escherichia coli*, *Stafilococcus aureus*, and *Candida albicans*) and exhibit controlled release of Ag^+^ ions, with generally low cytotoxicity levels. The most frequent applications included catheters, dental prostheses, dressings, and orthodontic resins. However, methodological heterogeneity and the scarcity of clinical trials limit the extrapolation of findings. Conclusions: AgNPs confer relevant therapeutic advantages to polymers, but long-term clinical studies are needed to confirm their safety and effectiveness.

## 1. Introduction

Medical device-associated infections (MDIs) represent one of the most pressing challenges in modern healthcare systems. It is estimated that between 25% and 50% of all nosocomial infections are directly related to the implantation of some type of biomaterial, whether an intravascular catheter, an orthopedic prosthesis, or an intraoral device [1,2]. According to the World Health Organization, such infections can increase patients’ hospital stays by up to 12 days and double the attributable mortality in vulnerable populations, resulting in additional global costs exceeding USD 7 billion annually [3]. This situation requires developing strategies to reduce microbial colonization while ensuring device functionality and safety [4].

In the field of dentistry, plastic polymers have proven to be highly useful materials due to their versatility, low cost, and favorable physicochemical properties [5]. Poly (methyl methacrylate) (PMMA), for example, is widely used in the fabrication of dental prostheses, splints, and denture bases due to its dimensional stability, acceptable aesthetics, and ease of handling [6,7]. Likewise, other polymers such as polyethylene and polyurethane are used in orthodontic devices, mouthguards, and components of clinical instruments due to their mechanical strength and adaptability to various manufacturing techniques [8,9]. These characteristics have established plastics as fundamental components in restorative, preventive, and surgical procedures. However, their surfaces can promote bacterial adhesion and biofilm formation, pose significant biosecurity challenges, and prompt the development of antimicrobial strategies to enhance their clinical performance [9,10,11].

Thermoplastic polymers—such as polyethylene (PE), polypropylene (PP), poly (methyl methacrylate) (PMMA), and polyurethane (PU), among others—dominate the medical device market due to their light weight, moldability, and compatibility with additive-manufacturing techniques. However, their chemically inert nature promotes the adsorption of serum proteins and, consequently, the formation of complex biofilms that can harbor multidrug-resistant pathogens such as *Staphylococcus aureus*. In response to this limitation, surface engineering has explored coatings with drugs, antimicrobial peptides, metal oxides, and, most notably, silver nanoparticles (AgNPs), the broad-spectrum activity of which encompasses Gram-positive and Gram-negative bacteria, fungi, and certain enveloped viruses [8,9,12,13,14,15,16,17].

AgNPs exert their bactericidal effect by generating reactive oxygen species (ROS), electrostatic interactions with the cell membrane, and the sustained release of Ag^+^ ions that disrupt DNA replication [18,19].

However, the widespread clinical adoption of this technology requires a rigorous evaluation of the biocompatibility, potential toxicity, and long-term behavior of AgNPs within plastic matrices [8]. Given the growing volume of literature and diverse methodologies employed, it is essential to conduct a systematic synthesis that identifies the quality of the evidence, estimates the magnitude of the antimicrobial effect, and clarifies the knowledge gaps that must be addressed in future research [20].

Consequently, in this comprehensive review, we aim to synthesize the available evidence on the antimicrobial efficacy of AgNP-functionalized plastics and identify the predominant therapeutic applications and the optimal concentrations of AgNPs.

## 2. Results

### 2.1. Search Results

The systematic search strategy enabled the identification of a total of 634 records distributed across five high-impact scientific databases: PubMed (*n* = 210), Scopus (*n* = 175), Web of Science (*n* = 120), SciELO (*n* = 52), and ScienceDirect (*n* = 77). After applying the inclusion and exclusion criteria defined in the methodology, 21 studies were selected for detailed analysis. These studies met the requirements for experimental design, antimicrobial evaluation, and characterization of silver nanoparticles (AgNPs) incorporated into plastic matrices for therapeutic purposes.

The distribution of the selected studies revealed a predominance of in vitro research (*n* = 15), focused on assessing antimicrobial activity, cellular cytotoxicity, and Ag^+^ ion release. These studies provide critical insights into the interaction between AgNPs and pathogenic microorganisms, as well as the biological compatibility of the functionalized materials.

In addition, four in vivo studies were identified, conducted primarily in animal models such as rats and rabbits. These studies offer evidence on the physiological effects of AgNPs in the contexts of wound healing, tissue regeneration, and the inflammatory response, although they present inherent limitations for clinical extrapolation [4,20,21,22,23].

Finally, two clinical trials were included, representing a significant advancement in the validation of these materials in real-world scenarios. One trial evaluated the use of dental resins containing AgNPs in patients undergoing orthodontic treatment, while the other analyzed AgNP-impregnated dressings applied to chronic wounds. Both studies reported clinical benefits in terms of microbial reduction, improved wound healing, and good patient tolerance [24,25].

The methodological diversity of the selected studies provides a comprehensive overview of the current state of research in this field, although it also presents challenges for the standardization of results. In this context, Table 1 summarizes the key characteristics of each study, including the type of polymer used, experimental design, physicochemical properties of the AgNPs, synthesis method employed, and the most relevant findings.

### 2.2. Qualitative Synthesis

The most used polymers were PU, PMMA, PE, PP, PLA, and PEEK [7,8,26,47,48,49]. In 20 out of 21 studies, a significant reduction in colony-forming units (CFUs) was observed against *Escherichia coli*, *Staphylococcus aureus*, and *Candida albicans*. Additionally, 10 studies reported the sustained release of Ag^+^ ions for at least 14 days.

Six studies included cytotoxicity assays using L929 fibroblasts, revealing cell viabilities above 80% when the concentration of AgNPs did not exceed 0.05% *w*/*w*. Clinical trials demonstrated reduced microbial colonization and shorter wound-healing times compared with controls [50].

### 2.3. Polymer Type Used

Polyurethane (PU) was the most frequently used polymer, applied in dressings, foams, and nanofibers. Its structural versatility and modifiability make it ideal for biomedical applications, and the incorporation of AgNPs significantly enhanced its antimicrobial and mechanical properties.

Polymethyl methacrylate (PMMA) was primarily used in dental prostheses and orthodontic resins. Studies such as Sun et al.’s [26] demonstrated that nanosilica functionalization improves AgNP dispersion without compromising the material’s aesthetics or mechanical strength.

Other polymers such as PLA, PEEK, HDPE, and PVP were also evaluated, showing positive results in terms of antimicrobial activity and cellular compatibility, although outcomes varied depending on the incorporation method and AgNPs’ concentration.

### 2.4. Microorganisms Evaluated

When integrated into polymeric matrices, these nanoparticles act as reservoirs, releasing the metallic ion in submicroscopic concentrations that inhibit microbial proliferation over extended periods (up to 90 days, according to in vitro studies) [51,52,53]. Unlike soluble antibiotic coatings, silver minimizes the emergence of resistant strains and can synergize with conventional therapies, reducing the required dose of systemic antibiotics [1,13,54,55,56].

The most evaluated microorganisms were as follows:

- Gram-negative bacteria: *Escherichia coli*, *Pseudomonas aeruginosa*;
- Gram-positive bacteria: *Staphylococcus aureus*;
- Fungi: *Candida albicans*.

In most studies, a ≥70% reduction in biofilms and bactericidal efficacy exceeding 99% were observed in AgNP-functionalized matrices. Some studies also reported activity against antibiotic-resistant strains, reinforcing the clinical potential of these materials.

### 2.5. AgNP Incorporation Methods

The methods used to incorporate silver nanoparticles varied across studies, directly influencing antimicrobial efficacy and biocompatibility, as follows:

- Electrospinning: Enabled the production of nanofibers with a high porosity and an active surface area, facilitating controlled Ag^+^ release.
- Direct impregnation: Used in PU foams; it demonstrated good nanoparticle retention and antimicrobial activity.
- Green synthesis: Applied to PLA and PVP, using plant extracts as reducing agents, with positive outcomes in microbial reduction and lower toxicity.
- Cathodic sputtering and chemical functionalization: Applied to polymers such as PEEK and HDPE, achieving homogeneous AgNP distribution and high bactericidal efficacy.

### 2.6. Observed Biological Effects

The most relevant biological effects were as follows:

- Cytocompatibility: AgNP concentrations of ≤0.05% *w*/*w* maintained >80% cell viability in L929 fibroblasts.
- Cell proliferation: Increased proliferation of fibroblasts and keratinocytes was observed in in vitro models.
- Cell migration and epithelialization: These processes were enhanced in the presence of AgNPs, promoting tissue regeneration.
- Accelerated wound healing: Reduced healing time, increased collagen deposition, and granulation tissue formation were documented in animal models.
- Anti-inflammatory and antioxidant effects: In vitro models have documented increased proliferation of fibroblasts and keratinocytes, as well as improved cell migration, resulting in faster epithelialization. In vivo studies, conducted primarily in rats and rabbits, have shown a significant reduction in healing time, along with increased collagen deposition and granulation tissue formation.

### 2.7. Comparison with Other Antimicrobial Strategies

Compared to conventional antimicrobial strategies, AgNP-functionalized plastics exhibit broader efficacy and reduced risk of microbial resistance. Agents such as chlorhexidine and triclosan provide targeted activity but are associated with cytotoxicity and resistance development. Antibiotics, while effective, act through specific pathways that bacteria can adapt to. In contrast, AgNPs exert multiple simultaneous mechanisms of action, including membrane disruption, reactive oxygen species generation, and protein/DNA interaction, which enhances antimicrobial efficacy. When embedded in polymer matrices, AgNPs generally demonstrate lower cytotoxicity at clinically relevant concentrations, although long-term biodistribution and systemic safety require further study.

### 2.8. Clinical Applications

The two clinical trials included evaluated the following:

- Dental resins containing AgNPs in patients with orthodontic appliances, showing reduced plaque accumulation and decreased gingival inflammation.
- AgNP-impregnated dressings in chronic wounds, with positive outcomes in healing, infection control, and clinical tolerance.

Although the results are promising, the need for multicenter clinical trials with a ≥12-month follow-up is emphasized to validate the safety and efficacy of these materials in real-world scenarios.

### 2.9. Advantages

The findings also demonstrate that the method of AgNPs’ incorporation significantly influences their efficacy. Techniques such as electrospinning enable the fabrication of nanofibers with a high porosity and an active surface area, facilitating controlled silver ion release. This sustained release not only prolongs antimicrobial activity but also reduces the risk of cellular toxicity, an essential consideration in dental applications where continuous contact with soft tissues occurs [10,13,30,56].

### 2.10. Limitations

Despite these advantages, concerns regarding cytotoxicity, genotoxicity, and potential dysbiosis have hindered widespread clinical adoption. Several studies have indicated that toxicity is critically dependent on particle size (<20 nm tends to penetrate biological barriers), concentration (>0.1% *w*/*w* may induce oxidative stress in fibroblasts), and surface coating (protein corona versus citrate or biocompatible polymers). Moreover, there is a growing debate about the environmental fate of AgNPs released during the device’s life cycle, as concentrations as low as 1 µg·L^−1^ can affect aquatic microbiota [1,57].

Although AgNP-functionalized plastics exhibit significant antimicrobial activity, their disposal raises concerns regarding environmental accumulation and ecotoxicity. Mitigation strategies include controlled incineration to prevent nanoparticle release, specialized recycling protocols to recover and neutralize AgNPs, and the development of biodegradable polymer matrices that minimize environmental persistence. Future research should also incorporate life cycle assessments to evaluate the ecological impact of AgNP-containing materials and establish standardized guidelines for safe and sustainable disposal.

### 2.11. Regulatory Considerations

The Food and Drug Administration (FDA) and the European Medicines Agency (EMA) require biocompatibility studies under ISO 10993-5 [58] for any biomaterial that releases silver, as well as ecotoxicity assessments. The lack of standardized testing protocols, combined with heterogeneity in synthesis methods (wet chemistry, laser ablation, or green synthesis using plant extracts), complicates cross-laboratory comparisons and the definition of safe-by-design concentrations [2,59,60,61,62].

Using the term “therapeutic” to describe AgNP-functionalized plastics requires precise conceptual delimitation. Although these materials demonstrate a significant capacity to reduce microbial load and prevent infections associated with medical and dental devices, their action is not directed at treating a specific disease, nor are they administered as direct patient interventions [63]. In this regard, their effect may be considered more prophylactic than therapeutic in the traditional clinical sense.

Although animal and in vitro models demonstrate promising antimicrobial and biocompatibility outcomes for AgNP-functionalized materials, advancing to large-scale human clinical trials requires careful ethical and technical consideration. Ethical aspects include obtaining informed consent, ensuring patient safety monitoring, and compliance with international bioethical guidelines. Technical requirements involve standardized dosing and exposure protocols, long-term follow-up to assess systemic biodistribution and toxicity, and strict adherence to ISO and FDA regulatory standards. Addressing these considerations will be critical to responsibly translating AgNP-functionalized plastics into clinical practice.

Moreover, classification as “therapeutic” carries important regulatory implications, as it may require robust clinical evidence, pharmacokinetic studies, and compliance with regulations applicable to active medical products. Therefore, it is recommended to use the term cautiously, accompanied by qualifiers such as “potential” or “complementary,” until sufficient clinical evidence is available to support their efficacy and safety in defined therapeutic contexts.

## 3. Discussion

In this comprehensive review, we aim to synthesize the available evidence regarding the therapeutic efficacy, biocompatibility, and safety of AgNP-functionalized plastics used in medical and dental contexts. A systematic search across five databases yielded 634 articles on the topic; however, only 21 met the inclusion criteria and were analyzed. Seven thematic categories were extracted from the literature: the type of polymer used, microorganisms evaluated, methods of AgNP incorporation, observed biological effects, clinical applications, advantages, and limitations.

The findings indicate that incorporating silver nanoparticles (AgNPs) into plastic matrices represents an effective strategy to enhance the antimicrobial performance of biomedical devices. Most of the included studies report a significant reduction in microbial load—particularly of *E. coli*, *S. aureus*, and *C. albicans*—as well as sustained Ag^+^ ion release, which contributes to prolonged bactericidal activity. Nevertheless, when these findings are contrasted with those of the seven key studies selected for critical analysis, both strengths and limitations emerge that must be considered for the clinical and regulatory validation of these materials.

Sun et al.’s study [64] stands out for its focus on PMMA functionalized with nanosilica (NS), which enhances AgNPs’ dispersion while preserving the polymer’s mechanical properties. The observed antibacterial activity and favorable cytocompatibility position it as a promising candidate for dental prostheses. However, the lack of clinical data and long-term ion release profiles limits its therapeutic extrapolation.

For their part, Sari et al. [45] propose a polyurethane (PU) foam combined with AgNPs and recombinant human epidermal growth factor (rhEGF), achieving accelerated wound healing in murine models of diabetic ulcers. This dual approach enhances both antimicrobial and regenerative activity, although questions remain regarding the systemic biodistribution of silver and its interaction with human tissues.

Choi et al.’s clinical study [28] provides direct evidence on the effectiveness of AgNP-functionalized TPU in diabetic patients, demonstrating a significant reduction in healing time and a lower incidence of postoperative infections. However, its retrospective design and lack of randomization introduce biases that should be addressed in future research.

Jamnongkan et al. [32] employed electrospinning to produce PLA nanofibers containing AgNPs, resulting in porous structures with high bactericidal efficacy. Although the thermal and mechanical data are encouraging, a decrease in cell viability was reported when silver concentrations exceeded 0.05% *w*/*w*, highlighting the need to optimize dosing to avoid cytotoxic effects.

In the sustainability field, Gómez-Lázaro et al. [36] presented an innovative approach based on recycled HDPE functionalized with 4-vinylpyridine and AgNPs. While in vitro results are promising, the absence of biocompatibility and long-term release studies prevents immediate clinical validation.

The studies by Wang et al. and Ahmed et al. [18,44], although non-experimental, offer relevant insights into the ecotoxicity of metallic nanoparticles. Wang et al. synthesized the environmental toxicity mechanisms of AgNPs, while Ahmed et al. demonstrated how morphology and chemical environment modulate toxicity in aquatic models. These and other studies reinforce the need to assess the environmental impact of silver-functionalized devices, particularly throughout their life cycle and final disposal [65].

Taken together, the analyzed studies support the therapeutic potential of AgNP-functionalized plastics, while also revealing significant methodological heterogeneity that hinders direct comparison across investigations. Variations in particle size (5–100 nm), concentration (0.01–0.5% *w*/*w*), synthesis method (wet chemistry, physical, and green), and polymer type (PMMA, PU, PP, and PE) prevent the establishment of standardized parameters for efficacy and safety.

The lack of standardized parameters for AgNP synthesis and polymer integration represents a major barrier to clinical translation. To address this, consensus guidelines should be developed for nanoparticle characterization, including size distribution, shape, zeta potential, and surface coating. Harmonized protocols for polymer integration and antimicrobial testing are also needed to ensure reproducibility across laboratories. Multicenter collaborations can strengthen validation, while regulatory agencies such as the FDA, EMA, and ISO should define safety thresholds and reporting requirements. Establishing such standards will enable more robust comparisons across studies and facilitate regulatory approval of AgNP-functionalized plastics for biomedical and dental applications.

Moreover, the scarcity of controlled clinical trials and long-term follow-up studies limits the regulatory validation of these materials. Most investigations focus on in vitro or animal models, with limited assessment of systemic toxicity, Ag^+^ ion pharmacokinetics, and potential cumulative adverse effects. Additionally, the environmental impact of nanoparticle release during device use and disposal remains a relevant concern yet is insufficiently addressed in the current literature.

The interpretation of findings was directly influenced by the methodological quality of the included studies. Most in vitro studies demonstrated a moderate risk of bias, primarily due to limited nanoparticle characterization and small sample sizes, which restricts the generalizability of their results. In vivo studies provided relevant insights into biocompatibility, but the absence of randomization and blinding reduced their reliability. Clinical trials, although ethically approved and clinically oriented, were constrained by small sample sizes and short follow-up periods. Taken together, these limitations led us to interpret the evidence with caution, highlighting promising trends while acknowledging that further high-quality clinical research is required to confirm the therapeutic potential of AgNPs in plastic matrices.

Therefore, future research should incorporate randomized, multicenter designs with ≥12-month follow-up, as well as ecotoxicity and ion release studies under simulated clinical conditions. The adoption of “safe-by-design” strategies—optimizing AgNPs’ size, coating, and concentration—could mitigate cytotoxic and environmental risks, facilitating their safe integration into medical devices.

In the dental field, these materials show promising applications in the fabrication of membranes for bone regeneration, coatings for surgical instruments, oral mucosa dressings, and drug delivery matrices. Although clinical studies remain limited, some registered trials have reported encouraging outcomes in the treatment of specific oral lesions.

It should be noted that the thresholds of ≥70% biofilm reduction and >99% bactericidal efficacy were reported directly in the included studies as indicators of significant antimicrobial activity. These values were not recalculated by the reviewers but were highlighted to reflect the common efficacy ranges described in the primary literature.

The substantial heterogeneity in particle size (5–100 nm), concentration (0.01–0.5% *w*/*w*), and synthesis methods (wet chemistry, green synthesis, physical methods) influenced the interpretation of findings. While smaller particles (0.1% *w*/*w*) tended to demonstrate stronger antimicrobial activity, these trends were not consistent across all studies, largely due to differences in synthesis protocols and incomplete reporting of nanoparticle characteristics. This heterogeneity limits the possibility of robust cross-study comparisons. To enable more reliable synthesis in future reviews, studies should systematically report physicochemical properties of AgNPs, including size distribution, shape, zeta potential, and surface coating.

## 4. Materials and Methods

### 4.1. Search Strategy

The bibliographic search strategy was conducted according to PRISMA 2020; it was designed to identify original studies evaluating the use of silver nanoparticles (AgNPs) incorporated into plastic matrices for therapeutic, medical, or dental purposes. Five high-impact, multidisciplinary scientific databases were selected: PubMed, Scopus, Web of Science, SciELO, and ScienceDirect. These platforms were chosen for their ability to index relevant biomedical, technological, and clinical literature, as well as for their compatibility with Boolean operators and controlled vocabulary.

The search period was defined from January 2013 to December 2024, aiming to cover a decade of scientific production that reflects both recent advances and consolidated trends in the development of AgNP-functionalized biomaterials. This timeframe allowed for the inclusion of studies employing modern methodologies for synthesis, characterization, biological evaluation, and emerging clinical trials.

Controlled vocabulary—such as MeSH descriptors in PubMed—and free-text keywords were used to ensure both sensitivity and specificity, adapted to the syntax of each database. The main terms included “silver nanoparticles,” “AgNPs,” “plastic,” “polymer,” “resin,” “therapeutic,” “medical,” and “antimicrobial.” These concepts were combined using Boolean operators OR and AND, enabling the construction of robust search equations that captured studies with diverse terminological and technical approaches.

In PubMed, the formula “Silver nanoparticles” [MeSH] AND plastic AND therapeutic* was used to retrieve studies employing standardized biomedical terminology. In Scopus, the equation TITLE-ABS-KEY (“AgNPs” AND plastic AND therapeutic*) focused on titles, abstracts, and keywords. In Web of Science, the expression TS = “silver nanoparticles” AND polymer AND antimicrobial was applied within the Topic Search field. SciELO—a database specializing in Ibero-American scientific literature—was explored using “Silver nanoparticles” AND plastic, while in ScienceDirect, the query “Silver nanoparticles” AND resin AND medical emphasized applied research articles.

For this review, inclusion criteria were defined to select original studies—whether in vitro, in vivo, or clinical—that incorporated silver nanoparticles (AgNPs) directly into plastic matrices for therapeutic purposes. Only studies reporting antimicrobial efficacy, silver release, or biological evaluation and published in English or Spanish were considered. Grey literature sources (such as theses, conference proceedings, and patents) were excluded to ensure methodological consistency and to focus exclusively on peer-reviewed evidence. Systematic reviews, meta-analyses, editorials, patents, studies lacking experimental results, and those using mixed metallic nanoparticles without a specific analysis of AgNPs were excluded, too. However, selected narrative reviews were cited only as contextual references to support the discussion.

The study selection was conducted in multiple stages. First, duplicates were removed using the Mendeley reference manager. Then, two reviewers independently assessed titles and abstracts, resolving discrepancies via consensus. Articles meeting the criteria were analyzed in full text, and the entire process was documented following the PRISMA framework.

A pilot form was designed for data extraction to collect detailed information on experimental design, polymer type, AgNPs’ size and concentration, synthesis methods, antimicrobial evaluation techniques, biocompatibility outcomes, and the main conclusions of each study.

The methodological quality of the included studies was assessed using specific tools according to study type: ToxRTool (Toxicological data reliability assessment tool) for in vitro research, the ARRIVE (Animal Research: Reporting of In Vivo Experiments) guidelines for in vivo studies, and the Cochrane RoB 2.0 tool for clinical trials. No meta-analysis was performed due to the heterogeneity of approaches and outcomes.

**Figure 1:**
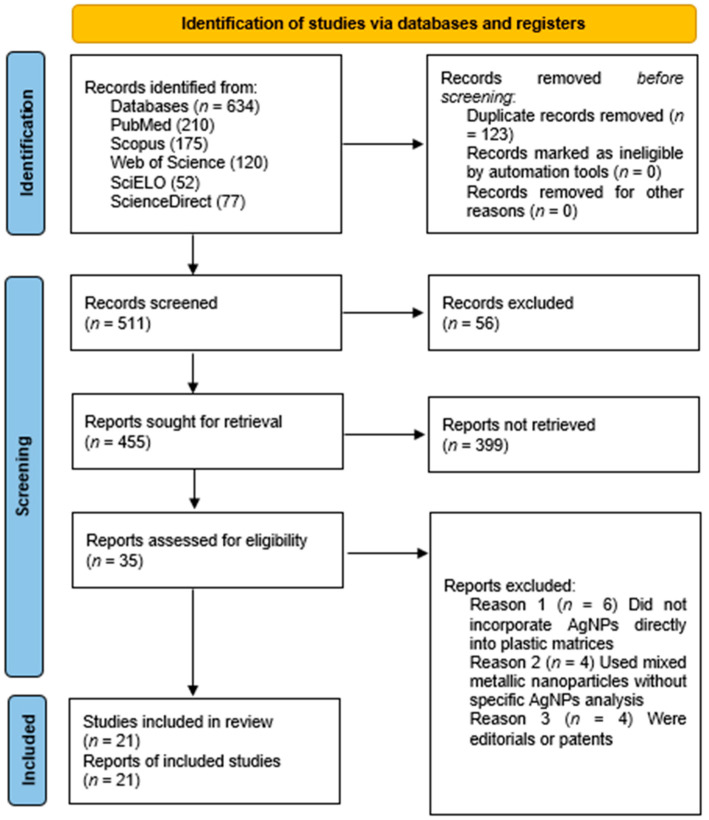
PRISMA 2020 flowchart of the study selection procedure from the reviewed articles.

Finally, the findings were organized narratively, grouping results according to polymer type and clinical application, to provide a clear and structured overview of the current landscape in the use of AgNPs in therapeutic plastic materials (Figure 1).

### 4.2. Search

Similarly, the strategy used to identify the relevant scientific literature on silver nanoparticles (AgNPs) in therapeutic and biomedical contexts—particularly in interaction with plastics, polymers, and resins—was documented using MeSH terms and Boolean operators (Table 2).

### 4.3. Risk-of-Bias Assessment

Following the description of study characteristics, a risk-of-bias and quality assessment was performed according to study type (in vitro, in vivo, and clinical), highlighting the main strengths, limitations, and overall risk of bias (Table 3).

### 4.4. Limitations and Strengths of This Study

This comprehensive review presents a structured and critical synthesis of the available scientific evidence on the use of silver nanoparticles (AgNPs) incorporated into plastic matrices for therapeutic purposes in medical and dental contexts. While the study provides relevant and up-to-date findings, it is important to acknowledge both its methodological strengths and the inherent limitations of its design and scope.

#### 4.4.1. Strengths

One of the main strengths of this work lies in the inclusion of five high-impact scientific databases—PubMed, Scopus, Web of Science, SciELO, and ScienceDirect—which enabled broad and multidisciplinary coverage of biomedical, technological, and clinical literature published between 2013 and 2024. This robust strategy facilitated the identification of relevant studies in both English and Spanish, enhancing the geographic and linguistic representativeness of the analyzed corpus.

This study also stands out for its multi-stage article selection process, with clearly defined inclusion criteria and specific tools for assessing methodological quality according to study type (ToxRTool, ARRIVE, and RoB 2.0). This differentiation allowed for a more accurate evaluation of internal validity in in vitro, in vivo, and clinical studies, minimizing interpretation bias and strengthening the results’ credibility.

Another notable strength is the findings’ thematic organization, grouped into seven analytical domains: polymer type, microorganisms evaluated, AgNP incorporation method, observed biological effects, clinical applications, advantages, and limitations. This narrative structure facilitated understanding of the current landscape, helped identify recurring patterns, and contributed to the formulation of specific recommendations for future research.

Additionally, this study incorporated a critical perspective on the use of the term “therapeutic” in relation to AgNP-functionalized plastics, acknowledging its regulatory implications and proposing a cautious conceptual delimitation. This reflection adds academic value by linking experimental evidence with ethical, normative, and semantic considerations relevant to clinical practice.

#### 4.4.2. Limitations

Nonetheless, this study also presents limitations that should be considered when interpreting its results. First, the methodological heterogeneity of the included studies—in terms of experimental design, polymer type, AgNPs’ size and concentration, synthesis methods, and the microorganisms evaluated—hinders direct comparison across investigations and limits the feasibility of conducting a meta-analysis. This variability prevents the establishment of standardized parameters for efficacy and safety, posing a challenge for clinical and regulatory validation.

Second, most of the analyzed studies correspond to in vitro or animal models, with limited representation of controlled and multicenter clinical trials. This limitation reduces the ability to extrapolate findings to real clinical scenarios, where factors such as immune response, the systemic biodistribution of nanoparticles, and interaction with human tissues may significantly alter material behavior.

Furthermore, a lack of uniformity was identified in protocols for evaluating cytotoxicity, ion release, and antimicrobial activity, complicating cross-study comparisons and the definition of safe concentration thresholds. In particular, the absence of data on Ag^+^ ion pharmacokinetics, cumulative effects, and systemic toxicity represents a critical gap in the current literature.

Another relevant limitation is the lack of attention given to AgNPs’ environmental impact, especially throughout the device’s life cycle and final disposal. Although some studies address ecotoxicity in aquatic models, most omit assessments of nanoparticle persistence in the environment, bioaccumulative potential, and risks associated with uncontrolled silver release in sensitive ecosystems.

Finally, although rigorous inclusion criteria were applied, some relevant studies may have been excluded due to language restrictions, limited access to full-text articles, or terminological variations not captured by the search strategy. This possibility should be acknowledged as an inherent limitation of any review process.

## 5. Conclusions

This review highlights the potential of silver nanoparticles (AgNPs) when incorporated into plastic matrices, particularly in medical and dental contexts. The analyzed studies show that this combination not only significantly enhances the antimicrobial properties of devices but also maintains acceptable levels of biocompatibility, provided that AgNP concentrations are properly adjusted. These findings open the door to promising clinical applications, where the prevention of device-associated infections is a priority.

Despite the observed advances, several aspects still require further attention. The current evidence, although encouraging, remains insufficient to establish definitive production standards or to guarantee the long-term safety of these materials. Moreover, the environmental impact resulting from silver release continues to be a relevant concern that must be addressed through targeted studies.

In this regard, AgNP-functionalized plastics emerge as a strong alternative for the development of safer, infection-resistant medical devices. To move forward, it is essential to promote multicenter clinical trials with extended durations and diverse patient populations to validate their efficacy in real-world scenarios. Likewise, it is crucial to incorporate ecotoxicological assessments into the design of new materials and to establish clear guidelines for good manufacturing practices that ensure these products’ reproducibility, quality, and sustainability.

AgNP-functionalized polyurethane exhibits significant potential for wound healing applications; however, translation into clinical practice faces several challenges. Systemic biodistribution and long-term toxicity remain critical concerns, as nanoparticles may accumulate in organs and tissues. Patient variability, lack of standardized dosing protocols, and the need for rigorous regulatory validation further complicate clinical implementation. Addressing these challenges through well-designed clinical trials and long-term safety assessments will be essential to ensure the safe and effective use of AgNP-functionalized polyurethane in biomedical applications.

## Figures and Tables

**Table 1:** Plastics used in combination with silver nanoparticles.

Nº	Author (Year)	Polymer	Type of Study	AgNPs: Size/Conc.	Synthesis Method	Key Findings
1	Sun J. et al. (2021) [26]	PMMA	In vitro + animal	~20 nm/0.05%	N/D	Biofilm reduction and good cytocompatibility
2	Ortiz-Magdaleno M. et al. (2023) [27]	PMMA	In vitro	15–30 nm/0.03%	Wet chemistry	Inhibits *S. mutans*, and mechanical properties are preserved
3	Choi H.-J. et al. (2017) [28]	PU foam	Animal	10–50 nm/ND	N/D	Improved wound healing in diabetic mice
4	Ahire, J.H. et al. (2024) [29]	PU nanofiber	In vitro	≤20 nm/0.01%	Electrospinning + loading	99% bacterial efficacy and sustained release
5	Zhang D. et al. (2022) [30]	TPU	Clinical study	N/D	N/D	Reduction in postoperative infection
6	Sienkiewicz N. and Członka S. (2022) [31]	PU foam	In vitro	N/D	Direct impregnation	>4 log10 reduction in *E. coli* and *S. aureus*
7	Jamnongkan T. et al. (202) [32]	PLA	In vitro	10–25 nm/0.02%	Electrospinning	Reduction of 5 log10 in 6 h
8	Samokhin Y. et al. (2025) [33]	PLA/CS	In vitro	N/D	Electrospinning	Antibacterial effect and good cell adhesion
9	Kahya N. et al. (2024) [34]	PVA/AgNW	In vitro	Nanothreads/ND	Physical	Photothermal and bactericidal effect
10	Lee S.J. et al. (2016) [35]	PEEK	In vitro	~30 nm	Cathodic sputtering	>99% bactericidal efficacy
11	Gómez-Lázaro B. et al. (2024) [36]	HDPE (high-density polyethylene)	In vitro	N/D	Chemical functionalization	High efficacy against *S. aureus*
12	Sofi H.S. et al. (2019) [37]	PU	in vitro	300 nm	N/D	Antibiofilm and cytocompatibility
13	Choi Y. et al. (2018) [38]	PLA/PU	In vitro	~25 nm/0.05%	Chemical reduction	High absorption and antimicrobial activity against *E. coli*
14	Soltanzadeh M.M. et al. (2024) [39]	PU	In vitro	N/D	Direct dispersion	Reduced adhesion of *E. coli*
15	Barik B. et al. (2024) [40]	RPO	In vitro	N/D	Green synthesis	Bacterial reduction
16	Liang W. (2023) [41]	Various	Review	–	–	Systematic summary of 45 studies
17	Sabarees G. (2022) [42]	Various	Review	-	N/D	Comprehensive review of recent advancements
18	Wang F. et al. (2024) [43]	PMMA	In vitro	~15 nm	Physical synthesis	Sustained Ag^+^ release and low toxicity
19	Ahmed I. et al. (2021) [44]	PVP + humic acid	In vitro	N/D	Green synthesis	5 log_10_ microbial reduction
20	Sari B.R. et al. (2021) [45]	PU	In vivo	N/D	N/D	Positive immunomodulation, rapid tissue regeneration
21	Gasga-Tapia V. et al. (2024) [46]	PU	Ecotoxicity	≤1 µg/L	Controlled release	Moderate environmental impact

**Table 2:** Summary of search.

Database	Date Range	Search Strategy	Records (*n*)
PubMed	2013–2024	“Silver nanoparticles” [MeSH] AND plastic AND therapeutic	210
Scopus	2013–2024	TITLE-ABS-KEY (“AgNPs” AND plastic AND therapeutic)	175
Web of Science	2013–2024	TS = (“silver nanoparticles” AND polymer AND antimicrobial)	120
SciELO	2013–2024	“Silver nanoparticles” AND plastic	52
ScienceDirect	2013–2024	“Silver nanoparticles” AND resin AND medical	77
Total			634

**Table 3:** Risk-of-bias assessment of included studies.

Study Type	Criteria Assessed	Main Strengths	Main Limitations	Overall Risk of Bias
In vitro (*n* = 15)	- Clear description of experimental design - Characterization of AgNPs (size, distribution) - Appropriate controls - Replicates reported	Most studies provided detailed methods and adequate controls	Some lacked standardized nanoparticle characterization, and small sample sizes	Moderate
In vivo (*n* = 4)	- Randomization of animals - Blinding of outcome assessment - Ethical approval - Reporting of adverse effects	Ethical approval reported; outcomes relevant to biocompatibility	Randomization/blinding rarely described; limited sample sizes	Moderate to high
Clinical trials (*n* = 2)	- Randomization - Allocation concealment - Blinding - Sample size justification - Outcome reporting	Both trials reported ethical approval and clinical relevance	Small sample sizes; limited follow-up; blinding unclear	Moderate

The risk-of-bias assessment was adapted to the study type. In vitro studies were evaluated for methodological rigor and nanoparticle characterization; in vivo studies for randomization, blinding, and ethical approval; and clinical trials for standard domains of bias (randomization, allocation concealment, blinding, sample size justification, outcome reporting). Overall, most studies presented a moderate risk of bias due to methodological heterogeneity and limited reporting.

## Data Availability

No new data were created or analyzed in this study.

## Abbreviations

The following abbreviations were used in this manuscript:

MDIs	Medical device-associated infections
PMMA	Polymethyl methacrylate
PE	Polyethylene
PP	Polypropylene
PU	Polyurethane
